# Use of Veterinary Vaccines for Livestock as a Strategy to Control Foodborne Parasitic Diseases

**DOI:** 10.3389/fcimb.2020.00288

**Published:** 2020-06-26

**Authors:** Valeria A. Sander, Edwin F. Sánchez López, Luisa Mendoza Morales, Victor A. Ramos Duarte, Mariana G. Corigliano, Marina Clemente

**Affiliations:** Laboratorio de Molecular Farming y Vacunas, Unidad Biotecnológica 6-UB6, INTECH, UNSAM-CONICET, Chascomús, Argentina

**Keywords:** protozoa, helminths, foodborne parasites, domestic livestock, animal health, veterinary vaccine

## Abstract

Foodborne diseases (FBDs) are a major concern worldwide since they are associated with high mortality and morbidity in the human population. Among the causative agents of FBDs, *Taenia solium, Echinococcus granulosus, Toxoplasma gondii, Cryptosporidium* spp., and *Trichinella spiralis* are listed in the top global risk ranking of foodborne parasites. One common feature between them is that they affect domestic livestock, encompassing an enormous risk to global food production and human health from farm to fork, infecting animals, and people either directly or indirectly. Several approaches have been employed to control FBDs caused by parasites, including veterinary vaccines for livestock. Veterinary vaccines against foodborne parasites not only improve the animal health by controlling animal infections but also contribute to increase public health by controlling an important source of FBDs. In the present review, we discuss the advances in the development of veterinary vaccines for domestic livestock as a strategy to control foodborne parasitic diseases.

## Introduction

**Figure 1:**
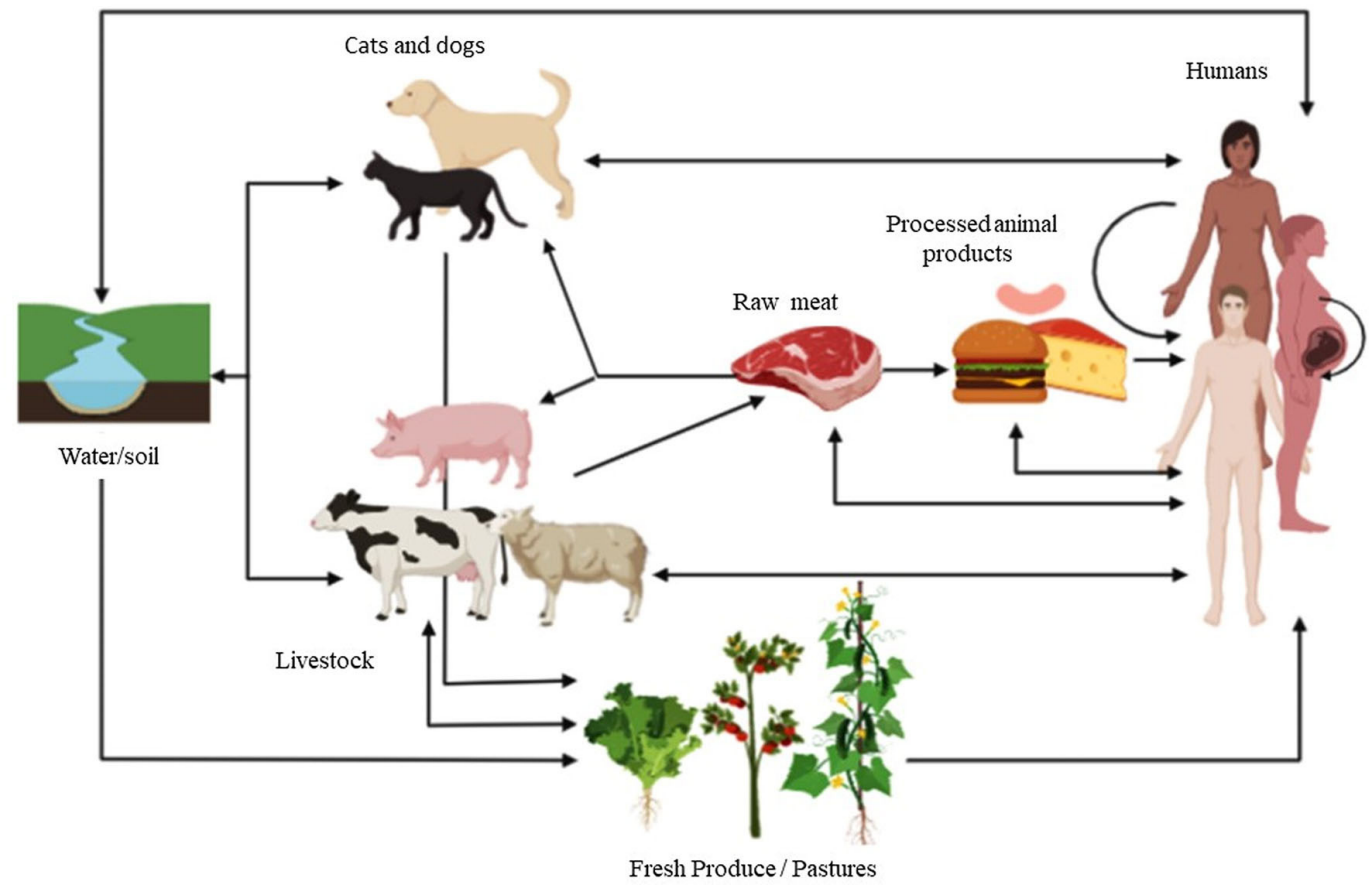
Livestock play a major role in the transmission of FBDs caused by parasites. They may be involved in the direct foodborne route of infection from animal to human via the consumption of raw meat (e.g., *T. solium, T. gondii, T. spiralis* infective form in meat), but they may also contribute to human infection through many other indirect and direct routes. Just to name a few, indirect routes include contamination of water or soil (e.g., *Cryptosporidium* spp. oocysts shed by cattle), contamination of fresh produce with eggs/oocysts (e.g., *Cryptosporidium* spp. oocysts shed by cattle), vertical transmission from mother to fetus after consumption of infected meat (e.g., *T. gondii*), infection of definitive hosts (companion animals such as cats and dogs) through the consumption of infected meat from livestock (e.g., *E. granulosus* infected sheep meat consumed by farm dogs) and among other direct routes, the human/animal contact represents an important one (e.g., *Cryptosporidium* spp and *T. spirallis* in farms and slaughterhouses sources). In the scheme, the arrowhead indicates the sense of the infection route.

Foodborne Diseases (FBDs) are a major cause of morbidity and mortality worldwide, affecting one-third of the global human population each year (World Health Organization, 2015a). Foodborne Diseases are caused by a broad range of chemical contaminants, bacteria, virus, parasites and biotoxins, and are often referred as neglected diseases. Despite parasites have not received the same level of attention as other foodborne biological and chemical hazards, they cause a high burden of disease in humans (World Health Organization, 2014). In fact, both Food and Agriculture Organization of the United Nations (FAO) and World Health Organization (WHO) have recently emphasized the global importance of foodborne parasitic diseases (Trevisan et al., 2019). Attending this issue, the FAO/WHO Foodborne Disease Epidemiology Reference Group (FERG) developed a multicriteria-based risk ranking of foodborne parasites at a global level (World Health Organization, 2014). Among the seven most important parasites listed, *Taenia solium, Echinococcus granulosus, Toxoplasma gondii, Cryptosporidium* spp., and *Trichinella spiralis* share a common feature: farm animals as important reservoirs (Devleesschauwer et al., 2017). This issue is of great relevance, not only to human health through the spread of FBDs and their consequences, but also to the production of food from animal origin because of the economic losses associated with affected livestock (Gajadhar et al., 2006; Newell et al., 2010). In this regard, the increase in human population and changes in consumer trends (more proteins in diet) will rise the consumption of animal products to 376 million tons by 2030 (Dhama et al., 2013). This huge demand on animal products, coupled with increasing concern about animal welfare, is prompting changes in farm-management leading to two different but potentially dangerous practices: (i) intensive animal management (Heredia and García, 2018) and (ii) preferences for animals raised outdoors (“organic animal management”) (Trevisan et al., 2019). While the intensive management and processing of products, with an increased movement of foods globally could lead to defective processing practices and, an augment of the risk of contamination by foodborne pathogens at any point of the farm to fork chain (Heredia and García, 2018), the “organic animal management” could lead to a greater possibility of infection with foodborne parasites (Trevisan et al., 2019). At this point, it is important to note that the pathways associated with transmission of foodborne pathogens to humans are complex and difficult to define, since the “direct” route from animal to man via meat is only one path, and humans may also infect one another directly, or contaminate meat during processing, etc. (Fegan and Jenson, 2018) (Figure 1). In fact, whereas *T. solium, T. gondii*, and *T. spiralis* represent a potential harm to human health mainly due to the consumption of undercooked meat or raw food derived from infected animals (Zolfaghari Emameh et al., 2018), *E. granulosus* and *Cryptosporidium* spp. are transmitted by other pathways, such as waterborne transmission, direct animal contact, and food contamination (Table 1).

**Table 1:** Main global ranked foodborne parasites that have livestock as an important reservoir and can be transmitted directly or indirectly to humans.

**Foodborne parasites**	**Human diseases**	**Main domestic and farm animal hosts^ς^**	**Food Category^ς^**	**Global ranking of foodborne parasites^§^**	DALYs^*^
*T. solium*	Cysticercosis (particularly neurocysticercosis) Taeniosis	Pigs	Pork Fresh produce	Ranked 1st	2,788,426 (2,137,613–3,606,582)
*E. granulosus*	Cystic echinococcosis.	Dogs, sheep and cattle	Fresh produce and water	Ranked 2nd	183,573 (88,082–1,590,846)
*T. gondii*	Toxoplasmosis. Congenital Toxoplasmosis.	Cats, sheep and pigs	Sheep meat and pork	Ranked 4th	1,153,779 (772,676–1,733,114)^+^; 526,515 (359,756–835,537)^#^
*Cryptosporidium* spp.	Cryptosporidiosis.	Cattle	Fresh produce and water	Ranked 5th	2,159,331 (1,392,438–3,686,925)
*T. spiralis*	Trichinellosis.	Pigs	Pork	Ranked 7th	550 (285–934)

ς *Taken from http://www.fao.org/3/a-i3649e.pdf*.

§ *Taken from Devleesschauwer et al. (2017)*.

* *Calculation of Disability Adjusted Life Years taken from Torgerson et al. (2015)*.

+ *Toxoplasmosis acquired*.

# *Congenital Toxoplasmosis*.

Epidemiological studies have helped to know the distribution of these foodborne parasites in domestic livestock and particularly, the role of cattle, sheep, and pigs in food contamination (Dhama et al., 2013; Torgerson et al., 2015; Rousseau et al., 2018). In fact, products of animal origin such as milk and meat from infected animals with these foodborne parasites are sources of contamination to other animals and humans (Davies, 2011; Fegan and Jenson, 2018) (Figure 1). In addition, livestock generate large volumes of feces, which can contaminate the environment with (oo)cysts or eggs, contributing to increase the prevalence of infections transmitted by some of these parasites in domestic animals (Fayer et al., 2010). In this sense, it is necessary to establish efficient strategies to control FBDs caused by these parasites in order to reduce the effects on animal and human health (Robertson et al., 2014). There are some generic good practices that are relevant for the control of foodborne parasites, as well as for other foodborne biological hazards, which have been reviewed by WHO-FERG (World Health Organization, 2014) which include control options enlisted as: (i) “primary production and pre-harvest measures” (on-farm measures, e.g., on-farm sanitation and hygiene); (ii) “post-harvest measures” (e.g., slaughtered practices and production procedures); and (iii) “education measures” (e.g., consumer education). In addition, specific guidelines to manage the most important foodborne parasites were also discussed, and include, between others, the used of chemotherapeutics and vaccines in livestock, when commercially available (World Health Organization, 2014).

In this regard, micro-environments on animal farms are usually ideal for long-term survival of parasite stages, such as eggs and oocysts, which are excreted by infected hosts (Gajadhar and Allen, 2004). The protective structure of the parasite in the exogenous stages (i.e., stages outside the host) allows many parasites to resist extreme temperatures, desiccation, and irradiation, making chemical methods commonly used for virus and bacteria control inefficient for preventing the spread of protozoa and helminths. Thus, the use of veterinary vaccines emerges as one of the most promising alternatives to control foodborne parasites in livestock, particularly in cattle, sheep, and pigs. This strategy not only would reduce transmission to humans (McManus and Dalton, 2006), but also the economic losses caused by the prevalence of these infections in livestock. In this review we will summarize the advances, future perspectives, and challenges in the development of veterinary vaccines for domestic livestock as a control strategy against FBDs caused by *T. solium, E. granulosus, T. gondii, Cryptosporidium* spp., and *T. spiralis*.

## Veterinary Vaccine Development Against FBDs Caused by Parasites

### General Considerations

Since its advent in the 1800s, vaccination remains, without doubt, the most effective medical intervention to prevent both, animal and human infectious diseases. Vaccination against zoonotic or foodborne infections is aimed at reducing or eliminating the risk for the consumer and, in some cases, to improve the productivity of the individual animal (Meeusen et al., 2007). It is generally accepted that the administration of vaccines for foodborne infections is the best-available public health intervention and, also represents the best cost-benefit ratio, occupying a preponderant place within the development millennium goals: to reduce mortality, to improve health; and to promote socio-economic development (Hewitson and Maizels, 2014; McAllister, 2014). In addition to the economic importance of these diseases, the difficulty of controlling them by chemotherapeutics methods and development of resistance against them, make the design of foodborne parasitic vaccines a relevant issue for human and animal health. In this regard, the development of antibiotic resistance will progressively reduce antibiotic therapy options available to veterinarians (Clifford et al., 2018). Therefore, vaccines would lessen the need for antibiotics to control diseases and offer veterinary practitioners much needed tools (Potter et al., 2008; Kolotilin et al., 2014). Moreover, the growing public interest in production of chemical-free food, which is fostered by consumer concern about drug residues in meat, eggs or milk supports the importance of the development of new veterinary vaccines (Murphy, 2012; Joachim, 2016).

As a strategy to control FBDs caused by parasites, veterinary vaccine development has practical advantages over human vaccine development, such as the ability to perform experiments in the natural host, therefore increasing its chances of achieving success compared to those of designed for humans, and the possibility to manufacture live-attenuated vaccines due to the lower safety requirements (Lightowlers et al., 2016; Zhang N. et al., 2018; Larrieu et al., 2019a). In fact, numerous vaccines against viral and bacterial infectious diseases have been produced by animal health companies and have been used for many decades as prophylaxis against pathogens in veterinary medicine (Lubroth et al., 2007; Jorge and Dellagostin, 2017). However, studies concerning vaccine design against foodborne parasites using livestock as animal models are scarce (Table 2). In fact, only few veterinary vaccines against these parasites are commercially available to be used in livestock, whereas there is none licensed for humans.

**Table 2:** Listing of results in vaccination against foodborne parasites targeted to domestic livestock applied as a single strategy.

**Foodborne parasite**	**Farm animal target**	**Vaccine type**	**Antigen + Adjuvant**	**Antigen delivery**	**Dose**	**Protection**	**Selected citation**
*T. solium*	Pigs	Recombinant protein	TSOL18 + QuilA	i.m. N.I.	200 μg 200 μg	Reduction in the number of cysticerci (99–100%) Reduction in the number of cysticerci (99.98%)	Flisser et al., 2004 Gonzalez et al., 2005
			TSOL16 + QuilA	i.m.	200 μg	Reduction in the number of cysticerci (99.8%).	Gauci et al., 2012
			TSOL45-1A + QuilA	i.m. N.I. i.m.	200 μg 200 μg 200 μg	Reduction in the number of cysticerci (97%). Reduction in the number of cysticerci (98.6%). No protection	Flisser et al., 2004 Gonzalez et al., 2005 (*) Gauci et al., 2012
			TSOL45-1B + QuilA	i.m.	200 μg	No protection	Gauci et al., 2012
			GK1, KETc1 and KETc12 + Saponin	s.c.	250 μg or 500 μg each antigen per piglet or sow, respectively	Reduction in the number of viable cysts (−97.9%). Reduction in porcinr cysticercosis prevalence (52.6%)	Huerta et al., 2001
		Bacteriophages	GK1, KETc1, KETc12 and KETc7	s.c.	4 × 10^11^ phages	Reduction in the number of cysticerci (52% non-viable cysticerci).	Manoutcharian et al., 2004
				oral	4 × 10^12^ phages	Reduction in the number of cysticerci (75% non-viable cysticerci)	
*E. granulosus*	Cattle	Recombinant protein	EG95 + QuilA	s.c.	300 μg	Reduction in the number of cyst (67–87%)	Heath et al., 2012b
			EG95 + QuilA	s.c.	300 μg	No protection	
			EG95 + QuilA	s.c.	0–150 μg	No protection	
			EG95 + Abamectin	s.c.	300 μg	No protection	
			EG95 + ISA 264	s.c.	300 μg	No protection	
			EG95 + ISA 773	s.c.	300 μg	No protection	
			EG95 + QuilA + ISA264	s.c.	300 μg	No protection	
			EG95 + QuilA + ISA773	s.c.	300 μg	Reduction in the number of cyst (71%)	
			EG95 + QuilA + To16/18	s.c.	250 μg	Reduction in number of cysts (88–99%)	
			EG95 + QuilA	s.c.	250 μg (to pregnant cows)	Reduction in the number of cysts in calves born from vaccinated cows (88–94%)	Heath et al., 2012a
			EG95 + QuilA	s.c.	250 μg (to pregnant cows) + 250 μg (to calves born from those cows)	Reduction in the number of cysts in calves born from vaccinated cows (87–100%)	
			EG95 + QuilA	s.c.	250 μg (to calves from unvaccinated pregnant cows)	Reduction in the number of cysts in calves (94–100%)	
	Sheep	Recombinant protein	EG95 + oil adjuvant EG95 + saponin EG95 + QuilA EG95 + ISA 70	s.c. i.m. + s.c s.c s.c.	50 μg 50 μg 50 μg 50 μg	Reduction in number of cysts (96%) Reduction in number of cysts (97%) Reduction in number of cysts (96%) Reduction in number of cysts (98%)	Lightowlers et al., 1996
			EG95 + ISA70 +saponin	s.c.	50 μg	Reduction in number of cysts (84.2%)	Poggio et al., 2016
			EG95 + adjuvant (N.I.)	s.c.	50 μg	Reduction of the infected 6-years old lambs from 56.3 to 21.3%. Reduction in the number of farms with infected lambs from 84.2 to 20.2%	Larrieu et al., 2019b
*T. gondii*	Sheep	Attenuated strain	Modified S48 strain tachyzoites (Toxovax® vaccine)	i.m	10^5^ tachyzoites (to ewes before mating)	Increased number of viable lambs (>70% in vaccinated ewes vs. 18% in non-vaccinated)	Buxton et al., 1991 Buxton, 1993
				s.c.	10^5^ tachyzoites (to ewes before mating)	Increased number of viable lambs (68%)	Buxton, 1993
			Mic1-3 KO strain tachyzoites	s.c. s.c. i.p.	10^5^ tachyzoites (to ewes before mating) 2 × 10^6^ tachyzoites (to ewes before mating) 10^5^ tachyzoites (to ewes before mating)	Increased number of viable lambs (62–91%) Increased number of viable lambs (71%) Increased number of viable lambs (92%)	Mévélec et al., 2010
		DNA	GRA1 + CpG + liposomes GRA4 + CpG + liposomes GRA6 + CpG + liposomes GRA7 + CpG + liposomes	i.m. i.m. i.m. i.m.	1 mg 1 mg 1 mg 1 mg	N.D N.D N.D N.D.	Hiszczynska-Sawicka et al., 2011a
			ROP1 + CpG	i.m.	150 μg, 300 μg, 400 μg, 600 μg,	N.D.	Li et al., 2010
			ROP1 + ovine CD154 ROP1	i.m. i.m.	1mg 1mg	N.D. N.D.	Hiszczynska-Sawicka et al., 2011b
			MIC3 + CpG + liposomes	i.m.	1 mg	N.D.	Hiszczynska-Sawicka et al., 2012
	Pigs	Live strain	RH tachyzoites	i.m.	1 × 10^6^ tachyzoites	Absence of clinical signs in challenged pigs. Reduction in parasite load	Dubey et al., 1991
			RH tachyzoites + IFA+ CpG	i.m.	4000 tachyzoites	Reduction in the number of infected pigs (>52%)	Kringel et al., 2004
		Protein extract	RH tachyzoites Crude rhoptry proteins + ISCOMs	i.m. s.c.	7 × 10^7^ tachyzoites 100 μg	Reduction in number of infected pigs (30%) Reduction in the number of infected pigs (20%)	García et al., 2005
			Rhoptry protein extract + QuilA	i.n.	200 μg	Reduction in the number of infected pigs (41.6%)	da Cunha et al., 2012
			Excreted–secreted antigens (ESA) + Freund	s.c.	2 mg	Reduction in the number of infected pigs (80%)	Wang et al., 2013
			Total lysate antigens (TLA) + QuilA	i.m.	500 μg	Reduction in the number of infected pigs (80%)	Rahman et al., 2019
		DNA	GRA1 and GRA7 + pJV2004 and pJV2005	i.d.	500 μg	Reduction in the number of infected pigs (70%)	Jongert et al., 2008
*C. parvum*	Cattle	Attenuated strain	Lyophilized oocysts	oral	5 × 10^6^ oocysts	Reduction in oocyst shedding and diarrhea	Harp and Goff, 1995
			γ-irradiated oocysts	i.m.	1 × 10^6^ oocysts	Reduction in oocyst shedding and diarrhea	Jenkins et al., 2004
		Recombinant protein	C7 protein (part of the P23 antigen) + TDM and MPL	s.c.	300 μg (administered to pregnant cows)	Reduction in oocyst shedding and diarrhea in calves fed with colostrum from vaccinated cows	Perryman et al., 1999
			P23 antigen + Freund	s.c.	300 μg	Reduction in oocyst shedding and delayed its onset	Askari et al., 2016
			Oocyst surface CP15/60 protein + oil adjuvant	s.c.	N.I.	N.D.	Burton et al., 2011
*T. spiralis*	Pigs	Protein extract	Partially purified stichosome antigens from the ML + Freund	i.p.	250–4,000 μg	Reduction in the recovery of adult and infective larvae (14–55%)	Murrell and Despommier, 1984
			Crude lysate of newborn larvae + Freund	i.p.	5 × 10^6^ newborn larvae	Protection against challenge infection (44–51%)	Marti et al., 1987

*The name given to the antigen in the study of Gonzalez et al. (2005) was TSOL45 whereas in the description of the used antigens, the authors cited that of Flisser et al. (2004), which corresponded to TSOL45-1A*.

*N.D, not determined; N.I, not informed; i.d, intradermal; i.m, intramuscular; i.n, intanasal; i.p, intraperitoneal; s.c, subcutaneous*.

A possible explanation for the scarce progress on vaccine development against foodborne parasites, could be due to scientific obstacles, such as the complexity and diversity of foodborne parasites and a poor understanding of the host/pathogen interactions (Hewitson and Maizels, 2014; Stutzer et al., 2018; Sander et al., 2019). These obstacles severely limit the objective selection of the elements to be considered in vaccine development, including the choice of the target species, the vaccine approach, the selection of antigen/s, the immune response to be targeted, the addition of adjuvants and the route of vaccination (Chambers et al., 2016). In addition, the implementation of vaccines against foodborne parasites in livestock is too expensive compared to chemical control, which is cheaper and easier to apply by farmers (Lightowlers et al., 2016). In fact, cost of development, practicality of use, challenges to licensing, and eventual market value are also crucial elements in the development of a commercial veterinary vaccine for livestock (Chambers et al., 2016). Despite these issues, the development of vaccines against foodborne parasites is still a significant research topic in medical and veterinary sciences.

Most of the veterinary vaccines evaluated in livestock against foodborne parasites belong to one of the following categories: live attenuated vaccines, killed vaccines, or subunit (and recombinant) vaccines (Table 2). Live-attenuated vaccines induce a strong humoral and cellular immune response, but their safety is questionable due to the risk of virulence reversion (Innes et al., 2011); while inactivated vaccines are safer and more stable than attenuated ones, but they are less potent and confer a weaker humoral immunity (Lee et al., 2012). On the other hand, recombinant subunit vaccines are easy to store, free of contaminants and proteolytic enzymes since they are chemically produced, and they are able to induce a protective immunity without toxic side effects or cross-linked immune reactions caused by other components present in the pathogenic organism (Nascimento and Leite, 2012). In addition, large-scale production and purification of a well-defined product can be achieved (Purcell et al., 2007). However, there are several limitations for vaccines based on the use of recombinant proteins, such as the proper choice of antigen/s, deficient immunogenicity, and poor cellular immune response (Gander, 2005; Blake et al., 2017; Sander et al., 2019). In addition, subunit vaccines do not have enough capacity to activate the innate immune response; therefore, they require the incorporation of some adjuvant into the vaccine formulation (Mohan et al., 2013; Sander et al., 2019).

In the following sections we will summarize and analyze the current veterinary vaccines against the previously mentioned foodborne parasites that have been evaluated in domestic livestock, as an attempt to find common features that should guide the selection of the elements to be included in future vaccine developments against these challenging pathogens.

### Taenia solium

The cestode *Taenia solium* is at the top of the global multicriteria-based ranking of foodborne parasites described by FAO/WHO (Devleesschauwer et al., 2017) (Table 1), and is the causative agent of cysticercosis in humans and pigs, leading to considerable health and economic burden (Dixon et al., 2020). In fact, *T. solium* neurocysticercosis is the most important preventable cause of acquired epilepsy, being responsible for more than 30% of the cases in endemic countries (World Health Organization, 2015b) and probably, in the world (Rajshekhar et al., 2006). In 2015, the FAO/WHO estimated that more than 50 million people is infected with this parasite worldwide, resulting in a considerable total of 2.8 million disability-adjusted life-years (DALYs) (World Health Organization, 2014). Humans are the only definitive host of *T. solium* and acquire taeniasis (the adult tapeworm infection) eating raw or undercooked pork containing cysticerci (the larval form of *T. solium*). In turn, they will shed eggs of *T. solium* in their stool, which if ingested by other (or the same) humans or pigs (the usual intermediate host) will lead to the development of cysticerci (Sánchez-Torres et al., 2019). Deficient levels of sanitation as well as pig husbandry practices and eating habits conditionate the prevalence of *T. solium* infection in a determinate region (World Health Organization, 2014).

Despite considerable efforts have been made to establish the optimal intervention for control and elimination of *T. solium*, a recent analysis by de Coster et al. (2018) has concluded that evidence on this issue is still limited. Several control strategies for *T. solium* have been evaluated so far, including vaccination of pigs and improvement in pig rearing and meat inspection practices (Okello and Thomas, 2017). However, currently cysticercosis control is mainly based on treatment of human and pig with anthelmintic drugs and health control measures through public education. Treatment of taeniasis in humans as a strategy to control the parasite burden is known as preventive chemotherapy, and is implemented as mass drug administration, targeted chemotherapy or selective chemotherapy; in mass drug administration the whole population of a predefined geographical area is treated at regular intervals, irrespective of clinical status; whereas targeted chemotherapy treats only specific risk groups at regular intervals and selective chemotherapy screens patients and subsequently treats according to clinical status (Okello and Thomas, 2017). Although these interventions have contributed to reduce the transmission of *T. solium*, cysticercosis has not been eradicated from endemic areas or where housing health conditions are ill-suited. The theoretical benefits of treating taeniasis on transmission of *T. solium* are clear; however, none of the researches carried out until now have included enough coverage of population or time-length to achieve a major and sustained reduction in *T. solium* transmission (Okello and Thomas, 2017). Hence, the practical effectiveness of this approach to control *T. solium* is scarce.

As mentioned above, pigs are the almost exclusive intermediate hosts of *T. solium* (Djurković-Djaković et al., 2013). Therefore, the development of a vaccine for pigs could contribute to interrupt the parasite's life cycle and eliminate the main source of infection for humans (Gonzalez et al., 2005). In fact, it has been suggested that vaccination of pigs in order to prevent the development of cysticerci would be considerably more cost-effective than vaccination of the potentially exposed human population (Hewitson and Maizels, 2014). Therefore, different larval antigens from *T. solium* have been studied as potential vaccine candidates (Kaur et al., 2020). In this sense, Flisser et al. (2004) designed a highly efficient recombinant vaccine based on an 18-kDa protein from *T. solium* oncospheres (TSOL18). Intramuscular vaccination of pigs with 200 μg of TSOL18 recombinant antigen + QuilA induces a high level of protection after the challenge with the parasite (Flisser et al., 2004; Gonzalez et al., 2005). Interestingly, independent experimental vaccine trials were conducted in Mexico, Peru, Cameroon and Honduras with similar promising results (Lightowlers, 2013). The assays demonstrated that TSOL18 vaccine is able to induce around 99–100% protection against an experimental challenge infection with *T. solium* eggs in pigs (Flisser et al., 2004; Gonzalez et al., 2005; Lightowlers, 2006). Later, Assana et al. (2010a) carried out field trials in Cameroon. The authors noted that the combination of TSOL18 vaccination together with a single oxfendazole treatment led to the complete elimination of *T. solium* transmission to pigs. In addition, they demonstrated that a TSOL18 vaccination schedule based on administration of the formulation at intervals of 3–4 months between the first and second immunization was effective in pigs (Assana et al., 2010b). In a more recent study, Poudel et al. (2019) demonstrated that the vaccination of pigs with Cysvax®, currently the first and only cysticercosis vaccine licensed for commercial production by Indian Immunologicals Ltd (World Health Organization, 2020), based on the TSOL18 antigen, together with medication with oxfendazole, undertaken at 3-monthly interval over a 10-month period in a *T. solium* endemic region of Nepal, eliminated the transmission of the helminth among the animals. These results suggest that the use of vaccines and chemotherapies together increases the chances of reducing the transmission rate of taeniasis/cysticercosis to humans through the consumption of pork, since drug treatment eliminated any existing infection in pigs while the vaccine prevented potential subsequent infection. However, the high costs of these treatment schemes, in addition to the fact that human is the definitive host of *T. solium*, make their implementation difficult. As an alternative approach, some authors suggested that the strategic drug-treatment of *T. solium* infection in humans, after a combined vaccine + drug intervention in the pig population may provide a fast and effective method for *T. solium* control (Assana et al., 2010b; Lightowlers, 2013; de Coster et al., 2018; Braae et al., 2019; Poudel et al., 2019). This strategy was recently supported by field studies by Okello et al. (2017) in the Southeast Asian area based on the application of two rounds of community mass drug administration with three consecutive doses of albendazole 400 mg at 6 months interval, combined with pig vaccination (TSOL18) and treatment (OXF), followed by a repeat pig treatment 1 month later for three iterations (first two combined with the human mass drug administration), which resulted in a significant decrease in human taeniasis and prevalence. Moreover, García et al. (2016) demonstrated that a similar strategy, based on human mass drug administration combined with pig mass drug administration and vaccination implemented in the entire rural region of Tumbes in Perú, achieved the interruption of transmission of taeniasis in 105 of 107 villages through a 1-year attack approach and elimination persisted in most areas for at least 1 year without further intervention. In addition, this strategy was also supported by Braae et al. (2019), based on the predictions of a mathematical/computational model, “cystiSim”, considering basic aspects of *T. solium* biology and a dynamic pig population, but, as other simulation studies, it has several limitations.

In addition to TSOL18, other recombinant proteins from *T. solium* oncospheres have been evaluated as candidate vaccine antigens in pigs, such as TSOL45-1A, TSOL45-1B (both protein isoforms of the family of related antigens designated as TSOL45) and TSOL16. Intramuscular vaccination of pigs with 200 μg of recombinant TSOL16, a protein associated with the penetration gland cells within *T. solium*, plus QuilA, conferred 99.8% protection in immunized animals against *T. solium* eggs challenge (Gauci et al., 2012), whereas results from TSOL45-1A were confusing. While the studies conducted by Flisser et al. (2004) and Gonzalez et al. (2005) showed that vaccination of pigs with this recombinant antigen conferred >97% protection after challenge with *T. solium* eggs, Gauci et al. (2012) demonstrated that TSOL45-1A did not provide statistically significant levels of protection against *T. solium* infection. On the other hand, intramuscular immunization of pigs with 200 μg of recombinant TSOL45-1B did not conferred protection against *T. solium* infection (Gauci et al., 2012).

In a different approach, three epitopes from *T. crassiceps* antigens that cross react with *T. solium* named GK1, KETc1, and KETc12 were used in the form of synthetic peptides as a three peptide-synthetic vaccine (S3P) against porcine cysticercosis. The subcutaneous immunization (250–500 μg of each peptide plus saponin) of pigs naturally exposed to the parasite during 12 months with S3P vaccine decreased the total number of *T. solium* viable cysts (97.9%) and reduced the prevalence of porcine cysticercosis (52.6%) in the field trial (Huerta et al., 2001). In an attempt to improve this vaccine, the three peptides (KETc1, KETc12, GK1) and a recombinant antigen KETc7 were expressed in bacteriophages at multiple copies. The pool of the four recombinant-heat inactivated phages induced high levels of protection against experimental cysticercosis in immunized pigs, evaluated as reduction in viable cysticerci (52% of non-viable cysticerci for subcutaneous immunization vs. 75% for oral immunization) (Manoutcharian et al., 2004). However, field trials are still needed to evaluate the effectiveness of the recombinant phage S3P vaccine (Manoutcharian et al., 2004). In this regard, a number of studies have also explored the expression of antigens from *T. solium* in edible plants (Monreal-Escalante et al., 2016) and the use of DNA-vaccines as an alternative approach with promising results, but these vaccines have not been optimally evaluated in pigs so far (Sciutto et al., 2007).

### Echinococcus granulosus

*Echinococcus granulosus*, is a small cestode from the Taeniidae family and the causal agent of cystic echinococcosis (CE), also known as hydatidosis, one of the most important zoonotic parasitic disease in humans worldwide (Pourseif et al., 2018). Unlike taeniasis/cysticercosis, which is endemic in rural areas in developing countries, CE is still prevalent in some regions of industrialized countries (Gajadhar et al., 2006). According to a recent WHO report, almost 3 million people are infected with *E. granulosus* worldwide, and that human CE prevalence is as high as 5–10% in endemic regions such as Argentina, Peru, Central Asia, China and East Africa. It is estimated that CE causes approximately 1 million DALYs annually, leading to an increasing public health and socio-economic concern in many areas of the world (World Health Organization, 2014).

*E. granulosus* requires two mammalian hosts to complete its life cycle. The definitive hosts are mainly canids, including the domestic dogs, which passes the segments of the adult parasite containing eggs or free eggs into the environment with its feces. The eggs are ingested by the intermediate host (many mammalian species, including sheep, goats, cattle and humans), in which larval stage and infectious elements develop and cause CE (World Health Organization, 2014). Despite CE is not considered to be “strictly” a foodborne disease, FAO/WHO experts suggested that it is mandatory to study its foodborne route, since it is one of the major contributors to the global burden of parasitic zoonosis (World Health Organization, 2014). In addition to its relevance to both, human and animal health, CE generates important losses in the livestock industry, affecting leather quality, milk production, and/or animal fertility. In this sense, the prevalence in livestock varies from 20 to 95% in slaughterhouses (World Health Organization, 2011).

Conventionally, the human treatment of CE relies on surgery and/or chemotherapy, which is based on 2 benzimidazole carbamates, mebendazole, and albendazole, which are the only anti-infective available drugs clinically efficient to avoid the larval growth of *Echinococcus* spp. (Wen et al., 2019). However, these drugs can cause severe side-effects, particularly in immunocompromised patients (Wen et al., 2019). Moreover, the mortality rate, among surgical cases, is about 2 to 4%, and increases if surgical and medical care are inadequate (World Health Organization, 2014). In order to control the life cycle of the parasite from dogs to humans or livestock, different strategies have been conducted, mostly by the use of anthelmintic drugs, promotion of slaughter hygiene and education (Craig et al., 2017; Larrieu et al., 2019a). A few CE control programs, based mainly in the administration of this drug to dogs, have been successful in insular countries such as New Zealand and Tasmania, but in continental endemic countries only moderate effectiveness in CE control have been made, probably because of the requirement to administer praziquantel to dogs in rural areas eight times per year over numerous years (Larrieu et al., 2019a). In this regard, Pourseif et al. (2018) suggested that, in comparison with chemotherapies, prophylactic vaccines may provide much more effective therapies against CE. Despite the vaccination of dogs (the main host for the prevalence of CE) may provide better clinical outcomes and may be much economical than vaccination of the intermediate hosts (sheep and humans), the population of homeless stray dogs in rural areas of endemic countries is not controlled; which results in a lesser interest for this particular vaccine development (Pourseif et al., 2018). The recent advent of a vaccine against *E. granulosus* targeted to sheep has demonstrated that vaccination of intermediate hosts of *E. granulosus* could reduce the level of parasite transmission and decrease the incidence of human infections (Larrieu et al., 2019a). Since sheep are the most important intermediate host for CE and the main source of meat in many countries in the world, some studies suggest that the combined strategy based on the vaccination of intermediate hosts (sheep) together with treatment of dogs with praziquantel would help decrease transmission of the disease to humans (Torgerson and Heath, 2003; Torgerson, 2006; Larrieu et al., 2019a).

Lightowlers et al. (1996) designed a recombinant vaccine based on a 45W antigen from the oncosphere of *E. granulosus* called EG95 and showed that this vaccine induces a high protection (around 96–98%) in sheep. Later, several studies were performed in experimental conditions to evaluate the potential of EG95 recombinant vaccine on the reduction of parasite transmission in sheep testing different concentrations of the antigen with different types of adjuvants (Poggio et al., 2016; Larrieu et al., 2019b). In addition, Larrieu and colleges have also assessed the impact of EG95 vaccine under field conditions (Larrieu et al., 2013, 2015, 2019b). Most of these studies were carried out in countries where the cystic echinococcosis is endemic, such as Argentina and Australia (Craig et al., 2017). Recently, Larrieu et al. (2019a) published a bibliographic review summarizing the successful cases of EG95 vaccine in sheep. This vaccine has shown to be highly effective even under field conditions. The results obtained are very promising, showing protection levels between 85 and 95%. Currently, EG95 vaccine has been registered for use in China and pilot programs have recently been conducted in Argentina, Chile, and Australia (Larrieu et al., 2019a). Given the effectiveness of EG95 vaccine in sheep and considering that CE has a high incidence in cattle, the EG95 vaccine was also assayed in cattle (Wen et al., 2019). Several studies have shown that immunization with EG95 vaccine induce a remarkable protective efficacy in bovine hosts against cystic echinococcosis (Heath et al., 2012a,b). In particular, high level of protection was afforded to calves from cows subcutaneously vaccinated with 250 μg of EG95 + QuilA after challenged them 9 or 17 weeks of age (Heath et al., 2012a). This recombinant vaccine has shown to be successful in cattle under experimental conditions; however, its effectiveness to be assessed under field conditions.

Despite EG95 vaccine has demonstrated to avoid infection in sheep after three doses during the first year of life suggesting that it would be a practical vaccine for implementation by livestock farmers; it is necessary to analyze different vaccination schedules and determine the effect on the reduction of infection in dogs and humans. In addition, the EG95 vaccine is very expensive, which limits its application to animals reared only in some of the most developed countries (Valizadeh et al., 2017). As an alternative vaccination strategy, a number of important antigen vaccine candidates targeted against the definitive hosts have been evaluated in dogs, including a 66-kDa fibrous protein called EgA31, which plays a key role in infection of humans and dogs (Petavy et al., 2008); a fatty acid-binding protein from *E. granulosus* called *E. granulosus* differential factor 1 (EgDf1), involved in the uptake, storage and transport of fatty acids in the parasite (Chabalgoity et al., 2000); and three proteins from the family of *E. granulosus* EgM, called EgM123, EgM4, and EgM9, potentially involved in egg maturation (Zhang et al., 2006; Zhang Z. Z. et al., 2018). Despite some promising results have been obtained in those preliminary studies, these results have not been confirmed yet.

### Toxoplasma gondii

The protozoan *Toxoplasma gondii* is one of the most successful parasites worldwide, capable of infecting all warm-blooded animals, including humans (Innes et al., 2011). Despite ~25–30 % of the world's human population is infected by *T. gondii* (Montoya and Remington, 2008), in the majority of immunocompetent individuals, toxoplasmosis is asymptomatic (Feustel et al., 2012). However, infection with *T. gondii* can cause serious disease in the developing fetus if pregnant women become infected for the first-time during gestation (Innes and Vermeulen, 2006). In addition, toxoplasmosis is also known for its severe sequelae in immunocompromised patients (Luft and Remington, 1992). In 2015, the FAO/WHO estimated that more than 20 million people illnesses are caused by acquired toxoplasmosis, whereas CT affects 500.000 humans worldwide, resulting in a total of 1.6 million DALYs (Torgerson et al., 2015). It was recently estimated that the total economic impact of foodborne toxoplasmosis is US $ 3,456 million, whose dominant component is directly related to healthcare costs (Scharff, 2012; Devleesschauwer et al., 2017). *T. gondii* is also considered a parasite of veterinary relevance, since CT and reproductive failures such as fetal death and abortions also occur in many other animal species, including livestock such as pigs, sheep and goats, in which it has been recognized as being responsible for major economic losses through abortions, stillbirths and neonatal mortality (Raeghi et al., 2011).

Regarding *T. gondii* life cycle, it can be divided into sexual replication, which occurs only in felids, including the domestic cat (definitive hosts), and the asexual replication, which takes place in all mammals and birds (intermediate hosts). Horizontal transmissions to humans are more frequently caused either by the ingestion of tissue cysts in infected meat or by the ingestion of soil, water, or food contaminated with sporulated oocysts derived from the environment, rather than by the direct infection from feline feces (Robert-Gangneux and Dardéc, 2012). In this regard, the ingestion of oocysts excreted by cats does not appear to pose a significant risk of infection in humans, given that oocysts are not infective when passed from cats to humans, and the duration of oocyst shedding is short (Kijlstra and Jongert, 2008; Petersen et al., 2010).

Despite many factors can affect seroprevalence in humans, infection rates in meat-producing animals play a major role, considering the relevance of the foodborne route involving the consumption of raw or undercooked meat. In this regard, sheep and goats are the main sources of infections to humans, and also represent the main hosts for this parasite in some countries (World Health Organization, 2014). The highest prevalence values have been observed in Europe (65 to 89% in adult sheep), while in the rest of the continents the prevalence in sheep is around 30% (Stelzer et al., 2019). On the other hand, the prevalence of toxoplasmosis in pigs is also high in some endemic areas (Djurković-Djaković et al., 2013). The presence of *T. gondii* in pigs is not only a source of infection for humans, but also an important cause of mortality in pigs, especially in neonatal ones (Dubey, 2009; Stelzer et al., 2019). Although raising pigs indoors in confinement has greatly reduced *T. gondii* infection in pigs, the recent trend of organic farming is likely to increase *T. gondii* infection in pigs again (Guo et al., 2015). In fact, recent estimations showed that pork consumption is responsible for around 12–15% of *T. gondii* infections in humans (Kijlstra and Jongert, 2008).

*Toxoplasma gondii* is a challenging parasite to control because of the large number of possible vehicles, both for foodborne and non-foodborne infections (World Health Organization, 2014). Control strategies against *T. gondii* based on education of high-risk consumers, particularly pregnant women, and immunocompromised individuals, are imperative (Innes and Vermeulen, 2006; Devleesschauwer et al., 2017). Despite the health concern in the human population and the economic losses in livestock associated with *T. gondii* infection, there is neither animal nor human treatment able to eliminate it from the host once the chronic infection has been established (Innes, 2010). Currently, the human chemotherapies against *T. gondii* are based in the administration of sulfadiazine and pyrimethamine (Daraprim) (Alday and Doggett, 2017). However, their side effect makes these treatments inefficient. Additionally, spiramycine is used in CT to treat infection during pregnancy, but its administration is still controversial, since multicenter studies conducted in Europe have shown conflicting results about its efficacy (Sander et al., 2018). Although prevention of the severe consequences of CT in pregnant women would be a major target for a human vaccine, there has been scarce progress on this issue, a possible reason may be the fact that CT is generally not considered a priority for public health (Innes et al., 2019). There are also scientific obstacles, including that the best protective results of vaccines against *T. gondii* have been observed with live vaccine preparations, but there are safety and regulatory issues that may impede their use in humans (Innes et al., 2011). Killed or subunit vaccines represent a more safer approach, but there challenges to overcome, including that they are generally associated with a poor cellular immune response, one of the most important features to get a proper protection against *T. gondii* infections (Sander et al., 2018, 2019). Recent advances in understanding the key protective immune responses may help to determine predictive algorithms for protective epitopes in order to design better subunit vaccines (Innes et al., 2019). In addition, as CT is similar in women and sheep, an animal model based in sheep may be helpful in progressing a vaccine against human CT (Innes and Vermeulen, 2006).

Regarding the control measures against *T. gondii* infection in livestock, as in the case of other foodborne parasites, it has been suggested that the combination of different approaches is the optimal strategy, including the implement of farm biosecurity protocols, hygienic measures and management practices. However, these control measures alone are not economically viable or completely effective, thus, it is necessary to additionally apply immune chemotherapeutic tools (Zhang N. et al., 2013). However, no safe and effective drug is currently available for toxoplasmosis in livestock. In fact, even if a drug were developed to eradicate the chronic infection in food-producing animals, its administration would represent a risk related to an increase in drug resistance and to drug residues entering in the food chain (Hiszczynska-Sawicka et al., 2014; Sánchez-Sánchez et al., 2018). Therefore, the development of a prophylactic treatment based on vaccination seems to be the most effective method to avoid the spread of the disease in livestock. Moreover, a vaccine against toxoplasmosis in farm animals would also help prevent infection to humans through safe meats reducing costs due to the rigorous following up of at-risk pregnant women, and control parasite reactivation in immunocompromised patients in order to avoid the use of toxic drugs during toxoplasmosis treatment (Mui et al., 2008). However, only one commercial vaccine is commercially available in UK, France, and New Zealand against *T. gondii*, and its use is limited to sheep (Sander et al., 2018). This live-attenuated vaccine (Toxovax®) was obtained from a modified strain (S48) of *T. gondii* originally isolated from an aborted lamb in New Zealand. By repeated passage in mice for many years, the strain lost the capacity to form tissue cysts and oocysts. Buxton et al. (1991) demonstrated that the vaccination of ewes before mating with S48 tachyzoites increases the number of viable lambs after experimental challenge with *T. gondii* oocysts during pregnancy. Additionally, it was demonstrated that one subcutaneous injection of this 2 ml suspension induces protective immunity for at least 18 months (Buxton, 1993). Despite the effectiveness of this vaccine, there are some controversial points that would explain why it has not been implemented in other countries (Sander et al., 2018). Toxovax® is a live-attenuated vaccine, which carries the risk of reverting to a pathogenic strain and causing disease, in humans and other hosts. In addition, Toxovax®, was unsuccessful in preventing toxoplasmosis in pigs, therefore different approaches in the development of vaccines against *T. gondii* have been conducted to reduce the incidence of toxoplasmosis in this species. Firstly, Dubey et al. (1991) demonstrated that immunization of pigs with 1 × 10^6^ tachyzoites of live *T. gondii* RH tachyzoites reduced parasite load in animal tissues. Later, Kringel et al. (2004) assessed whether the addition of a CpG motif to 4000 RH tachyzoites enhanced protection levels. The results showed that ~52% of the examined tissue from vaccinated pigs was free of *T. gondii* (Kringel et al., 2004). In addition, immunizations were also performed using different fractions of *T. gondii* protein extracts. García et al. (2005) evaluated subcutaneous vaccination of pigs with 100 μg of *T. gondii* crude rhoptry proteins in ISCOMs. Although the immunization was able to induce a humoral response against the parasite, only 20% of vaccinated animals were free of tissue cysts (García et al., 2005). Later, da Cunha et al. (2012) analyzed intranasal immunizations with 200 μg of *T. gondii* rhoptry proteins plus QuilA. The authors observed a 41.6% of protection against challenge infection in pigs. More recently, Wang et al. (2013) revealed that the subcutaneous immunization with 2 mg of tachyzoite-pooled excreted–secreted antigens (ESA) supplemented with Freund's adjuvant was capable of reducing the levels of cysts in the muscle. Tissue cysts were not detected in 80% of immunized pigs (Wang et al., 2013). In a more recent study, Rahman et al. (2019) evaluated the efficacy of intramuscular immunization of pigs with 500 μg of total lysate antigens (TLA) from *T. gondii*. The TLA + QuilA vaccine induced a strong immune response and reduced the parasite DNA load below the detection limit in most pigs. Finally, recombinant antigens have been evaluated by DNA vaccines. Jongert et al. (2008) evaluated whether intradermal DNA vaccination with GRA1 and GRA7 proteins from dense granules was able to generate immune responses and to protect against tissue cyst formation in pigs. The authors demonstrated that administration of 500 μg of a cocktail DNA vaccine + pJV2004 and pJV2005 as adjuvants is able to elicit humoral and cellular immune responses against *T. gondii* in pigs and the protection achieved was around 70%. Similarly, efforts are being made to find a safer vaccine formulation for sheep, and this is clearly due to the significant economic and welfare impacts that CT has on the sheep livestock worldwide (Innes et al., 2019). In addition, this species may also be a more relevant animal model than the mouse to test potential human vaccines against CT, as the disease in pregnant sheep and pregnant women has similarities, as previously mentioned (Innes and Vermeulen, 2006). Most research in sheep have focused on the use of recombinant DNA-based vaccines (Li et al., 2010; Hiszczynska-Sawicka et al., 2011a,b, 2012). The evaluated antigens belong to the protein families of dense granules (GRA1, GRA4, GRA6, and GRA7; Hiszczynska-Sawicka et al., 2011a), ropthries (ROP1; Li et al., 2010; Hiszczynska-Sawicka et al., 2011b) and micronemes (MIC3; Hiszczynska-Sawicka et al., 2012). As with other foodborne pathogens, there are few studies that have shown conclusive results in livestock. Although all these studies have demonstrated that the evaluated antigens are capable of inducing a specific immune response in vaccinated sheep, the protective capacity of these recombinant vaccines remains to be elucidated. More promising results were obtained by Mévélec et al. (2010). They demonstrated that a Mic1-3 knockout *T. gondii* strain is able to prevent the abortion in sheep. A dose of 10^5^ Mic1-3 knockout tachyzoites was sufficient to induce protection after both subcutaneous and intraperitoneal injections. This experimental trial showed that Mic1-3KO vaccine increase the rate of viable lambs (62–91%) from vaccinated Bizet ewes (Mévélec et al., 2010). Despite the promising results, no new studies were reported to validate the effectiveness of mutant non-cyst-forming-based vaccine formulations in sheep.

The other area for a vaccination approach is the strategic vaccination of cats in order to reduce environmental contamination through a decrease of oocyst-shedding (Innes et al., 2019). However, a recent modeling study by Bonačić Marinović et al. (2019) showed that prospects on preventing oocyst-originated human toxoplasmosis by vaccination in large populations of cats are not favorable due to the large vaccination coverage needed, and that this vaccine approach might only be effective if applied in small cat populations such as those present in farms. Despite these discouraging conclusions, a few researches in vaccine development against *T. gondii* infection targeted to cats have been done, including the subcutaneous immunization with live Co-irradiated tachyzoites of *T. gondii* Beverley strain (Omata et al., 1996), oral immunization with ME49 strain tissue cysts (Freyre et al., 2007) or with *T. gondii* T-263 strain cysts (Frenkel et al., 1991) or bradyzoites (Freyre et al., 1993; Mateus-Pinilla et al., 1999). Additionally, subunit vaccines including as antigens ROP2 (Mishima et al., 2002; Zulpo et al., 2017) or crude rhoptry proteins (García et al., 2007; Zulpo et al., 2012) were also tested. However, with the exception of T-263 strain vaccine, none of the previously mentioned formulations completely prevented oocyst-shedding in the tested animals. T-263 strain vaccine showed promising results, but several issues related to the large-scale production, distribution and safety of the vaccine resulted in the avoidance of its commercial production (Choromanski et al., 1995). More recently, Ramakrishnan et al. (2019) used a CRISPR/Cas9 strategy to engineer a *T. gondii* strain with defective fertilization and decreased fecundity that is able to generate oocysts which fail to produce sporozoites. The immunization of cats with this engineered parasite strain totally prevented oocyst excretion following infection with *T. gondii*. Although these results are highly encouraging, a vaccine candidate consisting of a genetically modified live parasite strain will not reach the commercial markets, at least in the short term, since the regulatory framework in most countries does not approve the use of these edited organisms yet.

### Cryptosporidium spp.

*Cryptosporidium* spp. are ubiquitous protozoan parasites, and are the causative agents of cryptosporidiosis, a gastrointestinal disease in a wide variety of vertebrate hosts, including humans and livestock (Hatam-Nahavandi et al., 2019). Cryptosporidiosis usually induces self-limiting diarrhea in immunocompetent individuals, but the infection can be severe and life-threatening, particularly in immunocompromised individuals and in infants (Khalil et al., 2018). In fact, in the 1980s, cryptosporidiosis was recognized as the major cause of chronic diarrhea in immunosuppressed patients (Current et al., 1983) and later, it was associated with childhood malnutrition and premature death in developing countries (Sallon et al., 1988). It was estimated by FAO/WHO that more than 64 million people are affected by cryptosporidiosis worldwide, accounting for more than 2.1 million DALYs (Torgerson et al., 2015). Particularly, *Cryptosporidium* is a leading cause of diarrhea morbidity and mortality in children younger than 5 years (Khalil et al., 2018). In this sense, a recent study by the Global Burden of Disease 2016 Stroke Collaborators (2019) estimated that in children under 5 years, cryptosporidiosis causes 4,224,000 DALYs, but increases to 12,868,500 DALYs after accounting for undernutrition associated DALYs.

*Cryptosporidium* spp. are monoxenous (complete life cycle in a single host) coccidian parasites, which lack of species-specificity, allowing cross-transmissibility between multiple hosts. The infectious stages of these parasites, the oocysts, are shed with the feces of their hosts in their fully infective form, with no external maturation required (Meinhardt et al., 1996). *Cryptosporidium* oocysts may survive and persist in the environment for long periods (Checkley et al., 2015). Routes of transmission include waterborne, person-to-person, zoonotic and foodborne (Devleesschauwer et al., 2017). Currently, *Cryptosporidium* spp. are considered the most important cause of waterborne diarrhea outbreaks worldwide (Checkley et al., 2015). Despite foodborne transmission of cryptosporidiosis is thought to be much less common than waterborne or person-to-person transmission, it is emerging as an important public health issue (Devleesschauwer et al., 2017). Over 20 *Cryptosporidium* species and genotypes have been identified in human patients; however, *C. hominis* and *C. parvum* are responsible for the majority of cryptosporidiosis reported in people worldwide (Ryan et al., 2016). In addition to the relevance of cryptosporidiosis as a human health concern, it is also one of the most important diseases in young ruminants, especially neonatal calves (Hatam-Nahavandi et al., 2019). The clinical presentation of *C. parvum* cause acute gastroenteritis disease in calves resulting in significant economic and production losses (Thomson et al., 2017). At this point, it is important to note that human and farm animal infections occur from consumption of food and water contaminated with oocysts presented in the feces of animals and humans. In addition, several reports suggest a zoonotic transmission of *Cryptosporidium* spp. from animals to humans due to simultaneous detection of *Cryptosporidium* in livestock and farmers and slaughterhouse workers (Xiao and Feng, 2008; Jafari et al., 2012; Firoozi et al., 2019). Therefore, livestock can play a major role as a source of human cryptosporidiosis since cross-contamination of raw meat with feces of animal in the slaughterhouses is a risk factor for human cryptosporidiosis (Xiao and Feng, 2008). Ramirez et al. (2004) reported a prevalence of *Cryptosporidium* spp. infection in US dairy farms as high as 95%, whereas in Europe, prevalence of 20–40% has been reported in young calves. It has been estimated that the worldwide annual excretion of *Cryptosporidium* spp. oocysts by livestock is as high as 3.2 × 10^23^, with cattle being the host species causing most environmental contamination, capable of carrying different *Cryptosporidium* species, including *C. hominis* which implies an associated significant public health risk (Hatam-Nahavandi et al., 2019).

Several measures have been proposed to control foodborne cryptosporidiosis, mostly directed to improve water quality throughout the water supply and food chain (Devleesschauwer et al., 2017). There are only few options to treat human cryptosporidiosis, since no vaccine is available and the only FDA-approved drug, nitazoxanide, does not provide benefit for malnourished children and immunocompromised patients with cryptosporidiosis (Ryan et al., 2016). Regarding chemotherapeutic treatments for livestock, the only licensed drug against cryptosporidiosis in calves is halofuginone lactate. Although halofuginone lactate can reduce oocyst shedding and the duration of diarrhea in calves, it is not capable of completely preventing or curing the disease (Lefay et al., 2001; Jarvie et al., 2005). In this scenario, the development of a veterinary vaccine for cattle against *Cryptosporidium* spp. has aroused great interest, since it not only would help prevent disease in calves and reduce oocyst shedding to the environment, but also would help decrease zoonotic transmission of cryptosporidiosis from cattle to humans (Innes et al., 2020). Different approaches have been tested in cattle to assess the potential of developing a vaccine against *C. parvum* (Thomson et al., 2017). Initially, immunization of newborn calves with killed (γ-irradiated or lyophilized) *C. parvum* oocysts (1 × 10^6^-5 × 10^6^ ocysts/ml) resulted in reduced oocyst shedding and diarrhea when compared to non-immunized calves (Harp and Goff, 1995; Jenkins et al., 2004). However, the vaccine did not prove to be efficacious when tested under field conditions (Harp and Goff, 1995). Considering that cryptosporidiosis mainly occurs in very young calves, it is important to take under consideration that it might be difficult to generate protective immunity against the parasite quickly enough through active vaccination (Innes et al., 2020). In this regard, a promising strategy is to immunize dams a few weeks prior to parturition to generate hyperimmune colostrum containing high titers of specific antibodies (Innes et al., 2011). This approach is not only beneficial to control disease in livestock but also for public health reducing environmental contamination with *Cryptosporidium* oocysts (Jenkins et al., 2004). Up to now, a few antigens have been explored as vaccine candidates against bovine cryptosporidiosis in pregnant cow. Perryman et al. (1999) evaluated the recombinant *C. parvum* C7 protein containing the C-terminal of the P23 antigen. Cattle were subcutaneously immunized three times at 2-week intervals with 300 μg recombinant C7 + trehalose dimycolate (TDM) and monophosphoryl lipid A (MPL) as adjuvants. The results showed that calves receiving the immune colostrum from dam immunized with this recombinant protein were protected against diarrhea and showed a significant reduction in oocyst shedding compared to control animals (Perryman et al., 1999). More recently, Askari et al. (2016) also evaluated the recombinant P23 protein as a vaccine for passive immunization of newborn calves. They demonstrated that administration of enriched colostrum from immunized dams with 300 μg of the recombinant protein emulsified with Freund's adjuvant inhibited over 90% the oocyst shedding by calves. Similarly, Burton et al. (2011) analyzed the antibody responses in calves fed with colostrum from dams vaccinated with a recombinant *C. parvum* oocyst surface CP15/60 protein. This study showed that calves had measurable quantities of the specific antibody in their serum, indicating that the passive immunity vaccine approach may be suitable to help prevent cryptosporidiosis in livestock (Burton et al., 2011). Although these developments showed partial success under experimental conditions, none of them was effective under field conditions (Innes et al., 2011; Mead, 2014).

On the other hand, the development of a vaccine against *Cryptosporidium* that prevents disease or reduces the severity of infection in humans is a relevant issue, particularly considering its consequences in early childhood in developing countries. However, a vaccine targeted to children in the first years of life brings several “extra challenges,” since in addition to the poor understanding of the host/parasite interactions and the uncertainties about the type of immune response that induces protective responses (a shared feature among foodborne parasites), vaccines administered to this group may have lower efficacy due to a number of reasons, including the young age of the child, interference by maternal antibodies, deficient nutrition, etc. (Mead, 2014). Thus, the successful design of a human vaccine against cryptosporidiosis still remains, without doubt, a long and winding road.

### Trichinella spiralis

The intracellular parasitic nematode *Tichinella spiralis*, can infect a wide number of carnivore and omnivore hosts, and is the major etiological agent of a zoonosis known as trichinellosis, regarded as an emerging and re-emerging disease in some parts of the world, particularly in Eastern Europe and Asia (Cuperlovic et al., 2005; Devleesschauwer et al., 2017). In humans, symptoms of trichinellosis range from nausea, diarrhea, and fever to more severe ones, including myalgia, myocarditis, and sometimes, death (Robertson et al., 2014). All infected animals, including humans, serve as both definitive hosts and potential intermediate hosts (Pozio, 2014). However, the human transmission of this disease is considered 100% foodborne and occurs mainly by the consumption of raw or undercooked meat and its derivatives from pigs contaminated with encysted first-stage larvae (Ortega-Pierres et al., 2015). Although the improvement in animal husbandry practices, meat inspection, consumer education, and medical care have contributed to prevent trichinellosis, it was recently reported that more than 11 million people are chronically infected with *T. spiralis* all over the world (Murrell and Pozio, 2011). In this regard, it was suggested that the risk of exposure to *Trichinella* is negligible for livestock produced under conditions of controlled management (Noeckler et al., 2019). However, traditional and domestic practices in pig farming are common in several regions, such as Eastern Europe and Argentina (Murrell, 2016), leading to an increasing concern about the risk of spillover of *T. spiralis* in these areas. On the other hand, *T. spiralis* can also cause huge economic burden to the livestock industry, mainly due to mandatory meat inspection in many countries ($ 0.12–$ 3.0/pig) (Pozio, 2015; Zhang N. et al., 2018).

The human treatment of trichinellosis is based on anthelmintic drugs and should be initiated within a few weeks after eating contaminated meat. As with other helminths, chemotherapeutic treatments are mostly based in the administration of mebendazole or albendazole. Although these drugs are described as relatively safe, they can induce bone marrow suppression. In addition, they cannot be used in pregnant women due to their teratogenic effects (Shimoni and Froom, 2015). Despite the control measures against trichinellosis are mainly based in controlled management of domestic pigs (Gamble et al., 2019) and “post-harvest measures,” including testing and processing methods, as well as consumer education (Noeckler et al., 2019), vaccination of pigs represents an alternative approach to trichinellosis control, especially for those pigs feeding under backyard or free-ranging conditions (Zhang N. et al., 2018). In fact, vaccination is able to give a lifelong protection, diminishing the use of chemical antiparasitic drugs (Zhang N. et al., 2018). During the last 30 years, several attempts have been made to develop an effective vaccine against trichinellosis. In this regard, early protection assays in pigs against *T. spiralis* were based in the administration of partially purified stichosoma antigens from the muscle larvae (ML) (Murrell and Despommier, 1984) or inactivated newborn larvae antigens (NBL) (total extracts) (Marti et al., 1987). Various concentrations were emulsified in Freund's complete adjuvant and injected intraperitoneally into the pigs. The stichosoma antigens induced only moderate protection (14–55%) mainly directed against the fecundity of female worms, whereas NBL inactivated vaccine was highly protective in swine (44–51%) against *T. spiralis* challenge (Marti et al., 1987). Despite these promising results, commercial production of an inactivated *T. spiralis* vaccine is economically non-viable. Thus, more recent studies have been focused on the identification of the best *T. spiralis* antigens that elicit an effective and safety enteral and systemic protection in the host. Several vaccine candidates based on single, multiple or total antigens from different stages of *T. spiralis*, used as crude protein extracts, recombinant proteins or as DNA vaccines, administered alone, or combined with adjuvants, as well as delivered by live carriers have been proposed (revised by Zhang N. et al., 2018). However, most of them were performed using the murine model, and have been focused in vaccine formulations capable of reducing the worm fecundity and / or decrease in muscle larval and adult burdens. In this regard, Bien et al. (2015) undertook a study to identify immunoreactive proteins from the ML and adult stages that are specifically recognized by anti-*Trichinella* antibodies and found a total of 18 proteins, 3 of them common for both adult and ML, including heat shock proteins, enolase and 5'-nucleotidase. Despite these proteins may be potential antigens for early diagnosis and the development of a vaccine against the parasite, further researches must be conducted. In view of the previous findings, the design of an effective vaccine against *T. spiralis* should include multi-epitopes/antigens from different life cycle stages and a proper adjuvant and/or delivery system and must be evaluated by different administration routes in the target species, the pig. These considerations limit advances in the development of a vaccine for veterinary use as a strategy to prevent trichinellosis.

## Conclusions, Future Perspectives, and Challenges

During the last decade, the spread of FBDs caused by parasites has led to an increasing concern. Indeed, a recent report of FERG has confirmed that foodborne parasites cause a high burden of disease in humans (Torgerson et al., 2015). While the need to reduce the burden of disease of these particular parasites is obvious, the best interventions to achieve this goal are not clearly defined. In fact, each of these parasites, as well as its associated FBD, has unique challenging characteristics, which turns the selection of the best control measure a very difficult task. Despite vaccination of farm animals undoubtedly improves animal health and reduces economic losses associated with livestock industries, the relevance of livestock vaccination as a measure to control foodborne parasitic diseases must be discussed for each FBD (regardless of whether it is meat-borne or not), since several factors including biological and technical criteria, as well as available resources, must be taken into account. Moreover, this intervention must also be weight depending on the geographical region it would be applied, considering its particular social and political conditions (Gabriël et al., 2018). In the case of *T. solium*, vaccination of pigs arises as one of the most promising strategies, and the best suitable option to control cysticercosis, mainly when combined with mass drug administration in humans (García et al., 2016; de Coster et al., 2018; Gabriël et al., 2018). Since transmission of the parasite is associated with social habits and hygiene, such as lack of use of sanitary facilities in farms, free-range pig husbandry, not rigorous meat inspection methods, and a lack of encouragement around safe consumption, interventions to improve this issues would be beneficial to control this disease, however; to alter these habits might take more than one generation (de Coster et al., 2018); thus these options might be considered as “long-term” measures. On the contrary, the vaccination of pigs combined with anthelmintic administration in humans has shown good results, leading even to the elimination of cysticercosis in an endemic area of Perú within a year (García et al., 2016), suggesting that it represents a potential successful “short-term” measure (García et al., 2016; Okello et al., 2017; Braae et al., 2019). However, vaccination with Cysvax®, the only commercially available vaccine based in the recombinant *E. coli*-expressed antigen TSOL18, requires two to three immunizations to animals, and a strict cold chain for the vaccine (de Coster et al., 2018), both items very difficult to attain in certain poor and remote areas. Thus, an interesting alternative that deserves to be considered in the development of new vaccine approaches against *T. solium* targeted to pigs is the recombinant expression of the highly immunogenic TSOL18 antigen (or others) in other platforms in order to produce mucosal or edible vaccines. In this regard, plants can be a valuable option to produce therapeutic proteins for animal health (Clemente, 2014; Sander et al., 2018). In fact, the expression of subunit vaccines in plants, especially for veterinary use, offer several major advantages when compared to conventional recombinant protein expression systems such as bacteria, yeasts, insect cells or mammals, including low-cost production by eliminating expensive fermentation and purification systems, scalability, ability to produce complex proteins, sterile delivery, cold storage/transportation and safety (Shahid and Daniell, 2016). Most importantly, they represent a versatile tool for the production of edible vaccines capable of eliciting immune responses in both mucosal and systemic tissues and protecting from pathogen invasion at the mucosal surfaces, thus contributing to the development of more efficient vaccines (Yácono et al., 2012; Albarracín et al., 2015; Fragoso et al., 2017; Rosales-Mendoza et al., 2018). Despite these advantages, it was not until recently that plant-edible vaccines have reached the commercial market (Concha et al., 2017). Thus, far, only few studies have explored this approach to develop a vaccine against *T. solium* in plants (Monreal-Escalante et al., 2016). Another element to be considered in order to improve vaccines in general, and the current TSOL 18 vaccine (considering the number of immunizations needed per animal), is the addition of more effective adjuvants. Despite the incorporation of adjuvants that significantly reduce antigen dose, enhance a broad range of immune responses and provide protection against pathogens or related diseases is a necessary trend for the development of more effective vaccines, research on new adjuvants is still a challenge in the development of vaccines against foodborne parasites. However, many molecules have demonstrated their ability to enhance and modulate the immune response, which could be incorporated into foodborne parasitic vaccines (Sander et al., 2019).

In the case of *E. granulosus*, whether immunization of domestic livestock (specially targeted to sheep) is the most suitable approach or not, is difficult to assess. Despite anthelmintic drug treatment of dogs (the definitive host) has been a successful tool to control CE in insular regions, it did not render similar results in continental rural areas, where homeless stray dogs are usually present and uncontrolled (Larrieu et al., 2019a). This limitation must be taken into account also when considering the development of a vaccine against *E. granulosus* for dogs. On the other side, despite CE is not a strictly foodborne disease since it is not meat-borne transmitted to humans, vaccination of sheep, the most important intermediate host, could reduce the level of parasite transmission and decrease the incidence of human infections (Larrieu et al., 2019a), especially when combined with the administration of anthelmintics to dogs (Torgerson and Heath, 2003; Torgerson, 2006; Larrieu et al., 2019a). Currently, the licensed vaccines against *E. granulosus* are based on the EG95 antigen, which has proven effective both for sheep and cattle (Heath et al., 2012a,b). An important alternative to explore is the development of multi-epitope veterinary vaccines, which would include potent antigens from both, the adult and larval stage of the parasite, and thus, could be applied to intermediate and definitive hosts. In addition, as previously discussed for *T. solium*, the establishment of new platforms for production of safe and highly immunogenic mucosal vaccines, despite highly encouraged has been scarcely explored (Chabalgoity et al., 2000; Petavy et al., 2008). Moreover, the development of new and safe adjuvants, in addition to the identification of novel protective vaccine antigens are also of high priority.

A different scenario is associated with FBDs caused by the parasites *T. gondii* and *Cryptosporidium* spp. Regarding *T. gondii*, as extensively discussed before, control strategies based on education of high-risk consumers (pregnant women and immunocompromised individuals) and vaccination of sheep (and other meat- producing animals), are generally accepted as some of the most appropriate interventions to control spread of toxoplasmosis in humans. In the case of *Cryptosporidium* spp., control measures are mostly directed to improve water quality throughout the water supply and food chain (Devleesschauwer et al., 2017). In this sense, the development of a veterinary vaccine for cattle against *Cryptosporidium* spp. not only would reduce oocyst shedding to the environment by calves improving, indirectly the water supply, but also would help decrease zoonotic transmission of cryptosporidiosis from cattle to humans (Innes et al., 2020). Despite their differences, these parasites shared, at least, two common features: i) there is no effective available commercial vaccine against neither of them; and ii) they are coccidian parasites. Successful vaccines against coccidian parasites are scarce and limited to the veterinary field and excluding Coxabic® (a subunit vaccine against chicken coccidiosis), they are based on live attenuated or whole killed organisms (McAllister, 2014). Despite the rational design of vaccines against coccidian parasites (and why not, foodborne parasites in general) has its own difficulties to overcome and represents singular challenges, there are some common features that should be taken into account when analyzing their feasibility. One of the main obstacles in the development of an effective vaccine against them is to elucidate the mechanisms used by coccidian parasites to escape the host's immune system (Wang et al., 2019). Therefore, it is imperative to shed light about the cellular and molecular processes that regulate the life cycle of these parasites in different intermediate and definitive hosts in order to identify new protective antigens and establish the mechanisms to monitor and optimize vaccine success. In fact, the new genomics, proteomics and transcriptomics techniques have allowed the identification of a broad spectrum of potential proteins that could serve as vaccine candidates for *T. gondii* (Sidik et al., 2016) and *Cryptosporidium* spp. (Di Cristina and Carruthers, 2018). An effective approach to better understand protein function is based in different gene-editing techniques to construct mutant strains of coccidian species (Suarez et al., 2017). Among them, the CRISPR/Cas9 technology has proven to be a powerful system, both for *T. gondii* and *Cryptosporidium* spp, to unveil the molecular and cellular biology of these parasites (Sidik et al., 2016; Di Cristina and Carruthers, 2018), which in turn, may lead to great progress in the field of vaccinology, with an increasing impact on animal health (Wang et al., 2019). As for other foodborne parasites, the development of novel adjuvants and better delivery systems (preferentially, development of mucosal and/or edible vaccines) will greatly improve vaccine formulations against coccidians. In this regard, the results obtained by the addition of the most promising plant-derived adjuvants for the development of coccidian vaccine formulations have been previously reviewed (Sander et al., 2019).

To assess the actual relevance of a vaccine against *T. spiralis* targeted to pigs as a control option to human trichinellosis is a very difficult issue. Undoubtedly, the development of this potential vaccine will help decrease the human burden of this disease (Zhang N. et al., 2018). However, considering that the risk of exposure to *T. spiralis* is negligible for livestock under conditions of controlled management, and that there are some specific “post-harvest measures” that should prevent human infection with this parasite through pork consumption, little attention has been paid to the development of such vaccine. The rational design of a veterinary vaccine against *T. spiralis* for pigs shares the main features previously discussed and highlighted for other foodborne parasites, such as search for more potent antigens, novel and safer adjuvants, appropriate delivery systems, etc.

It is noteworthy that vaccination is a safe tool that would also help reduce resistance by the misuse of antibiotics and other drugs. Therefore, it is important to generate public education strategies to emphasize the importance of vaccination and its benefits. Public health agencies are responsible for properly informing about the importance of developing new vaccine formulations, including new adjuvants, and educating the population in order to increase compression and acceptance. In the last decades, many efforts have been focused on the development of effective immunoprevention against different foodborne pathologies. Progress to date on vaccination against parasites in livestock have provided evidence that may contribute to the development of more effective vaccines against the most important foodborne parasitic diseases and strongly impact on human health. To conclude, whereas the control of FBDs caused by parasites will be facilitated by the development and administration of veterinary vaccines for livestock, the achievement of this goal will require more coordinated and better dialogues between a broad spectrum of actors and stakeholders, to design and implement control measures that take into account scientific, cultural, educational, economic and political factors to tackle these neglected diseases.

## Author Contributions

MC and VS provided the ideas and wrote the draft manuscript. ES, LM, VR, MGC contributed to the editing, and revision of the manuscript. All the authors read the final manuscript.

## Conflict of Interest

The authors declare that the research was conducted in the absence of any commercial or financial relationships that could be construed as a potential conflict of interest.
